# Prevalence and Distribution of Developmental Defects of Enamel in Children Aged 12–15 Years in Fazilka District, Punjab, India, and Their Correlation With Drinking Water Fluoride Level

**DOI:** 10.7759/cureus.51238

**Published:** 2023-12-28

**Authors:** Sandeep Sidhu, Navneet Kathuria, Bela Mahajan, Gagandeep K Sidhu, Karthikeyan Ramalingam

**Affiliations:** 1 Oral Pathology and Microbiology, Maharaja Ganga Singh Dental College and Research Centre, Sri Ganganagar, IND; 2 Oral Pathology and Microbiology, Institute of Dental Sciences, Jammu, IND; 3 Oral Pathology and Microbiology, Saveetha Dental College and Hospitals, Saveetha Institute of Medical and Technical Sciences, Saveetha University, Chennai, IND

**Keywords:** esthetics, enamel hypoplasia, dde index, fluoride concentration, dental fluorosis, enamel defects

## Abstract

Background and objectives: Dental fluorosis is a developmental disturbance of dental enamels, caused by successive exposures to high concentrations of fluoride during odontogenesis, leading to enamels with lower mineral content and increased porosity. The objective of the present study was to assess the prevalence and severity of developmental defects and their relationship to fluoride levels in drinking water.

Methods: Ten villages were selected from Fazilka district, Punjab, India. A total of 1000 (519 males, 481 females) school children aged 12-15 years formed the study population. Eutech ION 2700 (Thermo Fisher Scientific, Waltham, Massachusetts, United States) was used for the estimation of fluoride levels in water. Developmental defects were screened and assessed using the modified Developmental Defects of Enamel (DDE) Index. Statistical evaluation was done using Karl Pearson's coefficient of correlation and the Chi-square test with IBM SPSS Statistics for Windows, Version 23, (Released 2015; IBM Corp., Armonk, New York, United States).

Results: The fluoride concentration in drinking water ranged from 0.5 to 2.0 ppm. The prevalence of developmental defects among the study population was 73.4% (range 59% to 100%). The most commonly observed type of defect was diffuse opacity (score 4) in 22.8% of the children. The premolars were the most commonly affected teeth. There was a significant positive correlation between the type (r=0.95; p<0.001) and extent (r=0.82; p<0.001) of developmental defects to the fluoride levels in drinking water.

Conclusion: The drinking water from about 50% of the villages had fluoride levels of 1 ppm or >1 ppm. A significant positive correlation between the severity of enamel defects and increased fluoride levels in water was deciphered. Thus, a simple, effective, and inexpensive method of de-fluoridation of drinking water should be prioritized if alternative sources of drinking water are not made available.

## Introduction

Tooth development is a well-coordinated process that is closely regulated by hereditary factors. Due to the extended duration of dentition development, it is very vulnerable to a wide range of environmental factors over an extended period [1]. Developmental defects of enamel refer to changes in the structure of enamel that may either impact a specific location or be more widespread, affecting all surfaces and layers of enamel. They may exhibit localization, generalization, and either symmetry or asymmetry. The causes of enamel defects may be either quantitative, resulting in insufficient enamel thickness, or qualitative, leading to enamel opacities [2].

The correlation between enamel defects and fluoride in drinking water has been proven during the last 75 years. Although dental fluorosis was prevalent in several regions of the United States, it remained unreported until Dr. Fredrick McKay saw the disease in Colorado in 1901. The local population referred to it as the Colorado stain. Dr. McKay had a keen interest in investigating this perplexing ailment, which he later called mottled enamel. The investigation into the role of fluoride in drinking water as a causative factor for mottled enamel or dental fluorosis was resolved by the research conducted by McKay et al. This research spanned from 1901 to 1931 [3].

Fluoride reaches the drinking water sources through the dissolution of fluoride-bearing rocks, soil, and surface dust. The common fluoride-bearing rocks are granites, granodiorites, schists, and grey wacks. From these sources, through drinking water and vegetation, fluoride finds its way into the human body [4]. Approximately two-thirds of a man's overall fluid consumption consists of water, while the remaining portion is derived from other nutritional sources. Depleted water from tissues must be replenished, and the replenishment quantities are mandatory. Nevertheless, in the case of developing children, the amount of fluid consumed may slightly exceed the amount of water lost, since there is a need for more water to facilitate the formation of new tissues [5]. In regions with a fluoride content of 0.1 parts per million (ppm) in drinking water, the concentration of fluoride in human rib bone reaches a plateau level of 2000 ppm when measured in dry, fat-free tissue. However, in locations where water is fluoridated at levels of 1.5 to 2 ppm, the plateau level of fluoride concentration in rib bones increases to 4000 ppm [6].

Dental fluorosis is a condition where the tooth enamel or dentin becomes hypomineralized due to the long-term consumption of high levels of fluoride during tooth development [7]. Dental defects provide enduring evidence of injuries sustained at certain stages of tooth formation. Fluorosis in dental enamel leads to the development of hypomineralization or porosity under the surface, which progresses toward the dentinoenamel junction as the severity of the condition worsens. The precise mechanism via which fluoride induces the alterations that result in enamel fluorosis remains unclear; however, it seems that the initial phase of enamel development is especially vulnerable to fluoride exposure. Dental fluorosis is significantly influenced by the amount, duration, and timing of fluoride exposure. The risk of enamel fluorosis is reduced when exposure is limited to the secretory stage, but it is maximized when exposure occurs during both the secretory and maturation stages [8]. The majority of observable abnormalities in the enamel of human teeth may be categorized using the Developmental Defects of Enamel (DDE) index into three kinds, depending on their visual characteristics: demarcated opacities, diffuse opacities, and hypoplasia [9,10]. There is a positive correlation between the level of fluoride exposure and the occurrence of enamel abnormalities in youngsters. In most circumstances, where there is not a significant amount of fluoride in the water, these defects are usually extremely less [7].

DDE may greatly affect both oral health and the appearance of teeth. Enamel defects are indicative of caries and erosion in children. Epidemiological studies consistently indicate an increasing prevalence of DDE in all groups, highlighting its clinical importance and providing data to support public health actions [9-12]. Understanding the epidemiology of enamel defects is crucial for informing the public about the developmental abnormalities of enamel and their connection to the fluoride concentration in drinking water. Additionally, it plays a role in evaluating and observing environmental and systemic elements, and maybe identifying the causative causes responsible for the formation of these enamel abnormalities. It is necessary to undertake such research across India to determine regions with elevated levels of fluoride and examine the methods and scope of defluoridation. This research aimed to evaluate the fluoride levels in the Fazilka region of Punjab, India, and correlate these levels with enamel defects of resident children.

## Materials and methods

We calculated the sample size using the following formula: n1= z2p(1−p)/e2. In the formula, n1 was the sample size, z was the level of confidence and when the confidence was 95%, z was 1.96, p was the prevalence of dental fluorosis and the non-response rate was 5%. The admissible error of prevalence was set at 15%. Finally, the required sample size was 960. For ease of documentation, we included 1000 samples for our study.

The current investigation was carried out on a total of 1000 pupils from 10 schools located in 10 villages of Fazilka district, Punjab, India. Data was gathered from a cross-sectional study that included examining enamel abnormalities in school children and estimating the fluoride content in the drinking water supply. A preliminary survey was conducted to provide an initial understanding of the number of children to be included in the study and the method to be used for measuring enamel hypoplasia. It was based on the fluoride mapping survey conducted by the Dental Council of India, the country's apex body for dental education.

Preliminary evaluations were used to ensure effective preparation and implementation of the primary investigation, as well as to determine the definitive format for data collection. The Institutional Human Ethics Committee of Surendera Dental College and Research Institute, Sri Ganganagar, Rajasthan, India, issued approval vide SDCRI/IHEC/22 toward the documentation of intra-oral findings.

Criteria for the selection of villages 

The research was carried out in ten villages located in the Fazilka area of Punjab. Fazilka is a recently established district among the 22 districts in the state of Punjab, located in the northwest region of India. There are a total of 314 revenue villages. The selection criteria for the research was that the village must have at least one high school and a reliable public drinking water supply sourced exclusively from groundwater. The demographic information of the villages included in the research was acquired from the corresponding Block Development Office. Before arranging the survey, we got formal authorization from the principals of the concerned schools of selected villages and parental agreement for the child's dental checkup. 

Criteria for the selection of samples

A pedagogical approach was used. The research included a sample group of children aged 12-15 who were enrolled in the local school located in the area being investigated. The reason for selecting children aged 12-15 is that by that age, all of their permanent teeth, except the third molars, would have fully emerged. This age group also has the additional benefit of being unlikely to exhibit behavioral and managerial difficulties, thereby resulting in improved cooperation. The survey only included individuals who had been inhabitants of the town for their whole lives.

The ultimate sample size was 1000 children selected from 10 schools located in 10 communities within the Fazilka area. Exclusion criteria were children undergoing fixed orthodontic treatment or with difficulty in the oral examination, or children who were absent from school during the investigation period.

Examination and collection of data

Enamel abnormalities were assessed before estimating fluoride levels to prevent any bias from the examiner. The examiners were postgraduate faculty in oral pathology who were trained by a senior faculty member (KR) with textbooks, relevant articles, and clinical images. Their competency was internally evaluated by KR before the initiation of the field study.

The investigators personally conducted all tests at the chosen schools. The examinations were administered during daylight hours, with the student positioned on a chair. A survey form was created and used to document the name, date of birth, gender, dietary preferences, and school affiliation of every kid. The pertinent data on the origin of the drinking water and the length of time the individual has lived at their current address were also collected. 

The presence of enamel hypoplasia was assessed using the modified DDE index (Clarkson and O’Mullane) [11] on the outer surfaces of six specific permanent teeth, namely the upper front teeth (central and lateral incisors) and the upper first premolars. 

A sterilized oral mirror was used to retract the cheeks or lips to enhance visibility. Teeth exhibiting severe tooth decay, restorations covering over 50% of the front surface, crown fractures affecting over 50% of the front surface, teeth that have not yet emerged, and flaws smaller than 1 mm were not included. The intra-examiner and inter-examiner repeatability was determined by re-evaluating 10% of the participants. 

Fluoride concentration estimation

Plastic bottles were used for collecting 500 ml of drinking water from the source, which were subsequently labeled and sent to the laboratory for fluoride analysis. Before collection, they were washed with the same water to be collected. The fluoride level of the concerned villages was obtained from the Water Testing Laboratory, Fazilka. Eutech ION 2700 (Thermo Fisher Scientific, Waltham, Massachusetts, United States) was used by the Water Testing Laboratory for the estimation of fluoride levels in water.

The collected data using the DDE index was tabulated and analyzed statistically. Karl Pearson's coefficient of correlation was used to analyze the data and determine the link between the fluoride levels in the drinking water and the kind and severity of developmental abnormalities. The Chi-Square test was used to determine the statistical significance using IBM SPSS Statistics for Windows, Version 23, (Released 2015; IBM Corp., Armonk, New York, United States) with a significance level of p <0.05.

## Results

**Figure 1:**
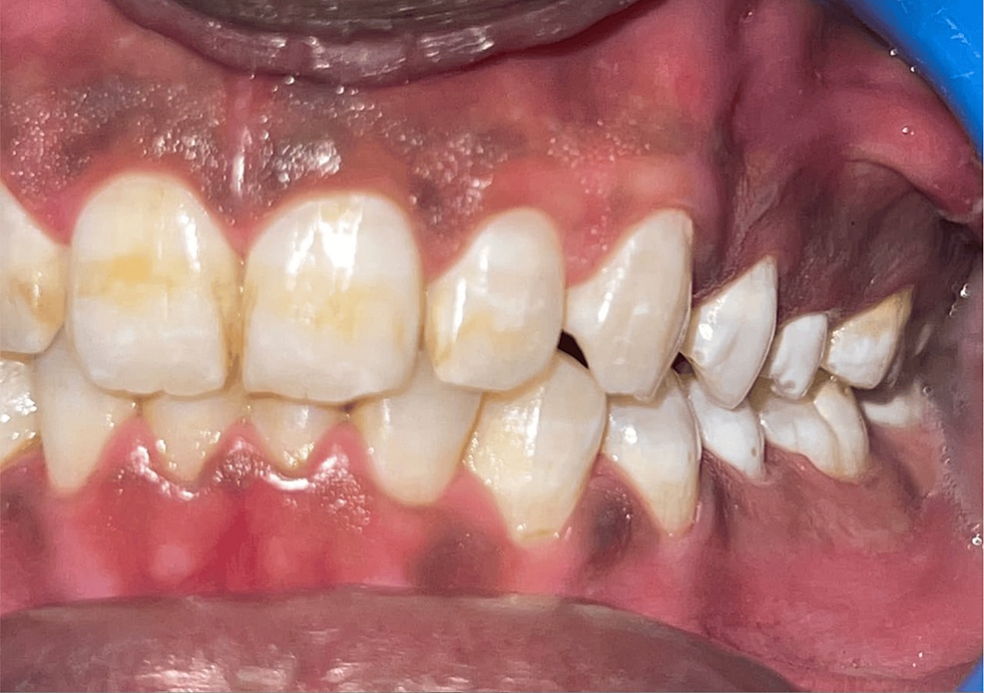
Clinical picture of dental fluorosis Intraoral picture showing diffuse mottling of enamel without any surface pitting.

**Figure 2:**
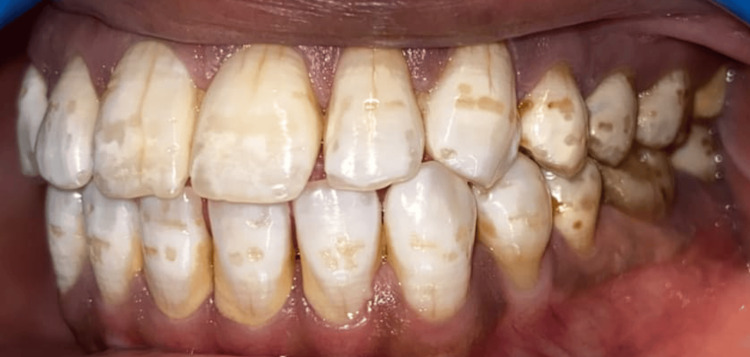
Clinical picture of dental fluorosis Intraoral picture showing dental fluorosis with mottling and pitting of enamel.

**Figure 3:**
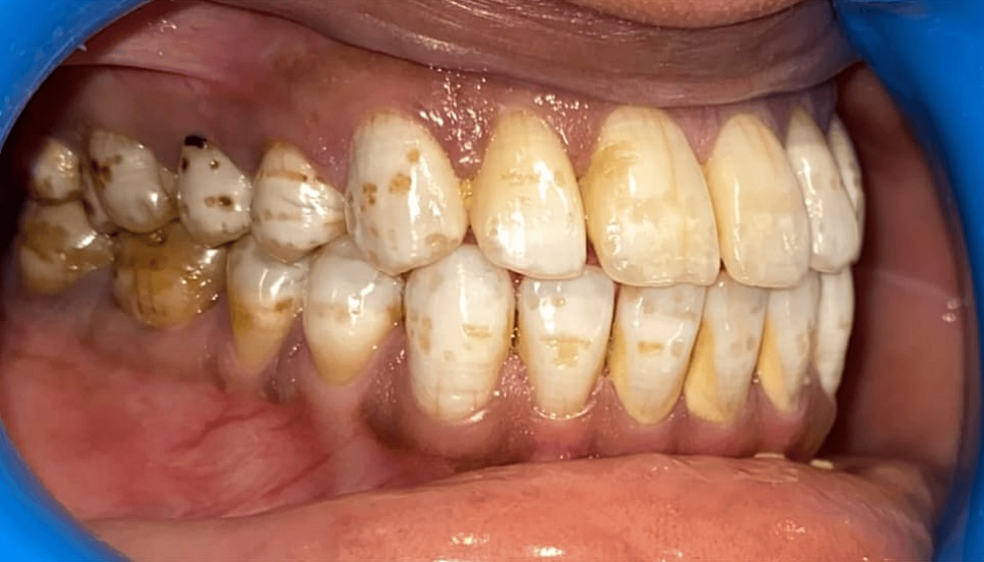
Clinical picture showing dental fluorosis Intra-oral picture showing diffuse mottling of enamel, pitting of enamel, and intrinsic stains.

We performed the study among various villages in the Fazilka District of Punjab. Dental fluorosis is identified as diffuse mottling of crown surface, mottling with surface pits, and mottling with surface pitting and staining as shown in Figures 1, 2, 3.

The prevalence of enamel defects ranged from 59% (n=59), 61% (n=61), and 67% (n=67) at 0.5 ppm to 97% (n=97) at 1.5 ppm and 100% (n=100) at 2.0 ppm of fluoride levels in drinking water. The number of affected and unaffected patients was statistically significant when the level of fluoride concentration increased. This prevalence concerning fluoride level was significant (χ2 = 82.52, df = 3, p < 0.0001). Table 1 reveals the prevalence of enamel defects correlating with fluoride levels.

**Table 1:** Data of the study samples The table gives details about the villages, their fluoride concentration, and the number of affected children with dental fluorosis. This prevalence concerning fluoride level was statistically significant (χ2 = 82.52, df = 3, p < 0.0001).

Study areas	Fluoride concentration in parts per million	Number of children examined	Affected by dental fluorosis	Unaffected
			n	n
Alam Shah	0.5	100	61	39
Ghurka	0.5	100	67	33
Pacca Chisty	0.5	100	59	41
Chak Tahli Wala	0.6	100	68	32
Behak Hasta Uttar	0.8	100	69	31
Behak Khas	1	100	71	29
Panchan Wali	1	100	73	27
Arrian Wala	1	100	69	31
Jhariwala	1.5	100	97	3
Rana	2	100	100	0
Total		1000	734	266

Table 2 represents the prevalence of enamel defects at < 1 ppm and at 1 ppm or > 1 ppm fluoride levels. The prevalence rate was 64.8% (n=324) at < 1 ppm and 82% (n=410) at 1 ppm or > 1 ppm. This difference in the observation was highly significant between enamel defects at low and high fluoride concentrations (χ2 = 37, df = 1, p < 0.0001).

**Table 2:** Table showing the fluoride levels and dental fluorosis The table shows that dental fluorosis was evident in 73.4% (n=734) of study samples even with lower levels of fluoride in the drinking water. This difference in the observation was highly significant between enamel defects at low and high fluoride concentrations (χ2 = 37, df = 1, p < 0.0001).

Fluoride level	< 1.0 ppm	1.0 ppm or	Total
		> 1.0 ppm	
Affected	324	410	734
Not affected	176	90	266
Total	500	500	1000
% Affected	64.80%	82%	73.40%

Table 3 demonstrates the total prevalence of the type of enamel defects (highest score in each affected student considered). Of the total sample size of 1000, 734 (73.4%) exhibited defects in the enamel, and 266 (26.6%) did not show any evidence of defects in the enamel. The distribution of the type of defects was as follows: 164 (16.4%) students showed demarcated opacities (white/cream), 92 (9.2%) - demarcated opacity (yellow/brown), 83 (8.3%) - diffuse (lines), 228 (22.8%) - diffuse (patchy), 51 (5.1%) - diffuse (confluent), and 95 (9.5%) - diffuse (confluent/patchy + staining + loss of enamel). Enamel hypoplasia was observed among the remaining 21 (2.1%) of the study subjects in the form of pits only. The value of r is 0.95. It means when fluoride concentration increases, the affected population also increases, indicating a very strong positive correlation. While code 0-type of defect shows the value of r = -0.95. It means when fluoride concentration increases, code 0 or normal or non-affected population decreases, indicating a very strong negative correlation.

**Table 3:** Table showing fluoride levels with types of enamel defects Table showing the fluoride concentration in different villages and its associated enamel defects along with Pearson correlation coefficient (r). The value of r is 0.95. This is a very strong positive correlation, which means that high X variable scores go with high Y variable scores (and vice versa).

Fluoride concentration in ppm	Study population	Affected population (n)	Type of enamel defect							
			0	1	2	3	4	5	6	7
0.5	100	61	39	23	5	8	17	3	5	0
0.5	100	67	33	21	9	6	19	7	5	0
0.5	100	59	41	27	7	5	15	1	4	0
0.6	100	68	32	20	10	7	22	2	7	0
0.8	100	69	31	21	8	11	19	2	8	0
1	100	71	29	15	16	5	23	7	5	0
1	100	73	27	18	16	4	19	6	10	0
1	100	69	31	19	21	3	13	3	10	0
1.5	100	97	3	0	0	17	39	11	22	8
2	100	100	0	0	0	17	42	9	19	13
TOTAL	1000	734	266	164	92	83	228	51	95	21
	Pearson correlation coefficient (r)	0.95	-0.95	-0.93	-0.35	0.71	0.86	0.73	0.89	0.9

**Figure 4:**
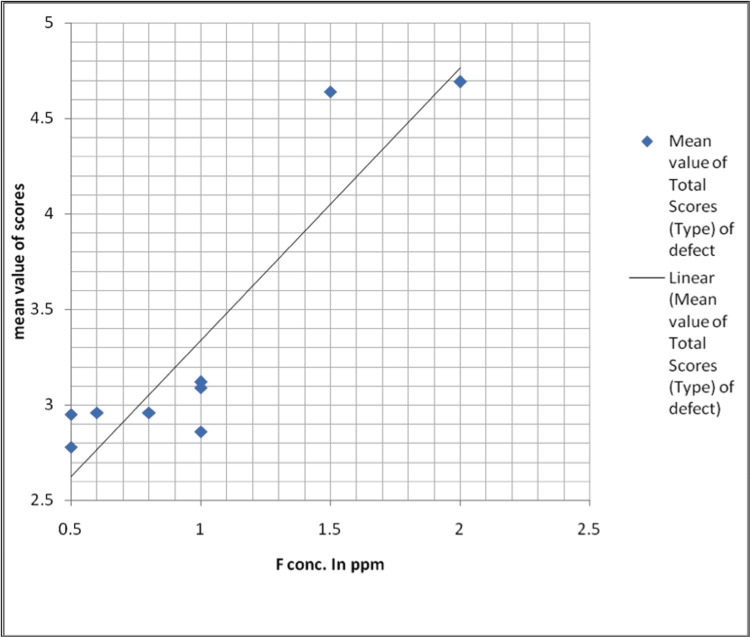
Graphical representation Graph showing the mean value of total scores based on the type of enamel defects between various fluoride concentrations in parts per million

The association between the overall severity of the type of enamel defects (mean value of total scores considered) with varying concentrations of fluoride in drinking water is shown in Table 4, Figure 4.

**Table 4:** Table showing the fluoride levels with mean value The table summarizes the fluoride concentration and its correlation with the mean value of the total score

Fluoride concentration in ppm	Study population	Affected population (n)	Mean value of total scores (type) of defect
				0.5	100	61	2.78
0.5	100	67	2.95
0.5	100	59	2.46
0.6	100	68	2.96
0.8	100	69	2.96
1	100	71	3.09
1	100	73	3.12
1	100	69	2.86
1.5	100	97	4.64
2	100	100	4.69

Distribution of enamel defects according to the extent was as follows: 233 (23.3%) cases had a score of 1 (< 1/3 facial surface), 248 (24.8%) cases had a score of 2 (1/3 to < 2/3), and 253 (25.3%) had at least 2/3 of the facial surface involved. The value of r was 0.82 with a strong positive correlation (Table 5).

**Table 5:** The extent of defects in dental fluorosis Table summarizing the fluoride levels with the extent of defects in dental fluorosis along with the Pearson correlation coefficient (r). The value of r is 0.82. This is a strong positive correlation, which means that high X variable scores go with high Y variable scores (and vice versa).

Fluoride concentration in ppm	Study population	Affected population (n)	Extent of defect			
			0	1	2	3
			n	n	n	n
0.5	100	61	39	28	18	15
0.5	100	67	33	31	14	22
0.5	100	59	41	20	16	23
0.6	100	68	32	17	31	20
0.8	100	69	31	23	30	16
1	100	71	29	33	25	13
1	100	73	27	30	32	11
1	100	69	31	28	21	20
1.5	100	97	3	21	35	41
2	100	100	0	2	26	72
TOTAL	1000	734	266	233	248	253
	Pearson correlation coefficient (r)	0.95	-0.95	-0.61	0.5	0.82

**Figure 5:**
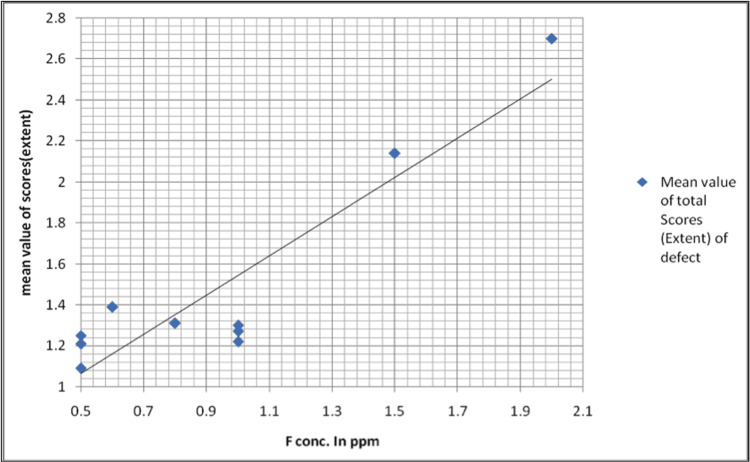
Graphical representation Graphical representation of the mean value of total scores based on the extent of defects and fluoride concentrations in parts per million. It shows the association between the overall severity of the extent of enamel defect (mean value of total scores considered) with varying concentrations of fluoride in drinking water.

The maximum extent of involvement of the tooth surface at < 1 ppm was <1/3rd in 23.8% and at 1 ppm or > 1 ppm of fluoride concentration was at least 2/3rd in 31.4% of the children. At < 1 ppm, score 1 was the most common, seen in n=119 (23.8%). At > 1 ppm, score 3 was the most common, seen in n=157 (31.4%). The prevalence of the extent of defects at low and high fluoride concentrations was statistically significant (χ2 = 8.48, df = 2, p = 0.0144). This observation was statistically significant (Figure 5).

## Discussion

In the current study, the concentration of fluoride in drinking water from the study areas of Fazilka district ranged from 0.5 ppm to 2.0 ppm. The overall prevalence of enamel defects in the present study conducted in the 10 villages of Fazilka district with a range of fluoride concentration between 0.5 to 2.0 ppm was 73.4% (range: 59% to 100%). Among the 10 villages studied, two villages had shown 97% and 100% prevalence, respectively. All villages showed a prevalence of over 50%. In their study, Rugg-Gunn et al. [12] examined three areas with fluoride levels of 0.22 ppm, 0.78 ppm, and 2.66 ppm, respectively. They discovered that 83% of the participants had one or more problems. The revised DDE index was used. In their investigation, Chandrashekar and Anuradha [13] discovered a prevalence of 69.4% with fluoride concentrations ranging from 0.22 ppm to 3.41 ppm. The research used the Community Fluorosis Index as its primary measure. Gopalakrishnan et al. [14] in a study conducted in one of the few areas of endemic fluorosis in Kerala, India, found a prevalence of 15.4% for fluoride concentrations ranging from 0.7 ppm to 1.4 ppm. Here the index used was Dean’s. In the study by Subba Reddy and Tewari [15], in rural areas of Bhatinda District, Punjab, which is a nearby district of Fazilka, the concentration ranged from 0.30 ppm to 10.40 ppm. At 0.30 ppm, the prevalence was 0.0%, whereas, at 1.10 ppm and 2 ppm, the prevalence was 85.02% and 98.05%, respectively. The index used was Dean's. In their research, Gayathri et al. [10] examined the frequency and intensity of developmental abnormalities and their correlation with the concentration of fluoride in drinking water. The fluoride content varied between 0.64 and 2.64 ppm. Developmental abnormalities were found in 88.5% of the cases. Our study indicated a greater occurrence of dental fluorosis in comparison to previously reported studies. The likely reason for this is the use of the DDE index in the research, which accounts for even the slightest abnormalities that may impact a single tooth. The results of most of the studies stated above are comparable to the results of the present study at similar fluoride levels. 

Very few studies utilized the modified DDE index for the prevalence of enamel defects according to fluoride level. The results of Rugg-Gunn et al. [12] are similar to those of the present study, while the results of Gayathri et al. [10] are on the higher side compared to the present study. Narbutaite et al. [16] reported the prevalence of dental fluorosis being 67.1% at 1.7 to 2.2 ppm and 3.7% at 0.16 to 0.18 ppm of fluoride concentration in drinking water. The prevalence of developmental defects at < 1ppm was 64.8% and at 1ppm or > 1ppm was 82%. This observation was statistically significant. The current investigation revealed a significant occurrence of enamel defects, even at low levels of fluoride concentration. In regions of India where the concentration of fluoride in drinking water is below 1 ppm, the occurrence and severity of dental fluorosis are more than anticipated [17,18]. 

Horowitz et al. [7] emphasized that the diagnosis of fluorosis, even in the absence of clear evidence of high fluoride sources, has been an often-seen occurrence. Studies have shown that when temperature rises, there is a corresponding rise in both the occurrence and severity of enamel fluorosis. This is attributed to the increased use of drinking water. Brouwer et al. [19] suggested that the elevated occurrence of enamel defects may be attributed to heightened perspiration and increased water consumption resulting from high daily temperatures and high altitudes. The impact of fluoride varied depending on climatic and lifestyle factors, with a more significant influence seen in places characterized by dry, hot, and arid environments, where health conditions may be uncertain. Nanda et al. [20] reported that Indian children consume a greater quantity of water. This phenomenon may be attributed to seasonal fluctuations, characterized by high temperatures in summer and chilly conditions during the monsoon and winter. Buildings offer little alteration in indoor temperatures. Furthermore, the level of fluoride in water was higher during the summer and monsoon seasons compared to winter. The fluoride concentration in water sources, as well as the amount of water used, exhibited seasonal variations. The values were higher during the summer season compared to the monsoon season, and higher during the monsoon season compared to the winter season. Therefore, it is likely that all of the causes indicated above might have contributed to the occurrence of developmental defects, even when fluoride is present in low concentrations. 

The current investigation found a strong statistical correlation between the concentration of fluoride and the severity and extent of the defect. As the fluoride concentration increased, both the kind and size of the defect also increased linearly. Among nine different types of enamel defects scored under the DDE index, the mildest defect found in the present study was demarcated opacity (white/cream) (16.4%), and the maximum severity was hypoplasia in the form of pits (2.1%). It was found that the most often seen defect was diffuse opacity (DDE Score 4), with an overall prevalence of 22.8%. The prevalence varied between 13% and 42% depending on the fluoride concentrations, which ranged from 0.5 to 2.0 ppm. Angelillo et al. [21], Nunn et al. [22], and Rugg-Gunn et al. [12] have also observed similar results in regions with different concentrations of fluoride in the drinking water. 

The current investigation found that the occurrence of diffuse opacity (score 4) at a concentration of 0.5 ppm was 17% and 19% in two specific regions. In another area, it was 22% at 0.6 ppm. Bardsen et al. [23] observed the presence of diffuse opacities in 33.3% of the children when exposed to low concentrations of fluoride in their drinking water (< 0.7 ppm). Warnakulasooriya et al. [24] have also documented similar results regarding dental fluorosis. It has been shown that there is a direct and proportional link between the amount of fluoride and the occurrence of dental fluorosis. Suckling and Pearce [25] found that the incidence of diffuse opacities rose dramatically with higher levels of fluoride exposure in drinking water. The dosage of fluoride played a crucial role in the development of dental fluorosis or diffuse opacities, together with the timing and length of exposure [8]. 

The present study showed Upper first premolars were the most frequently affected teeth in the present study with a prevalence of 35.42%. Upper central incisors and upper lateral incisors showed prevalence of 33.92% and 30.66%. Bardsen et al [23] reported that dental fluorosis is more prevalent in the maxillary teeth than in the corresponding mandibular teeth in the permanent dentition. They found that the premolars in both the maxilla and mandible were most often afflicted, followed by the second molars. Narbutaite et al. [16] found that premolars and second molars are the teeth most often impacted. The aforementioned teeth were chosen based on research indicating their heightened vulnerability to abnormalities. Limiting the examination to index teeth proved to be a more efficient and time-saving approach, while still providing valuable data on public health and cosmetic conditions. The examination focused only on the buccal surfaces since these surfaces have a greater frequency of abnormalities and are also the only areas that are cosmetically significant. 

Many studies have not included the defects on premolar and molar teeth in overall prevalence data. This is because they have examined children below the age of 12 years including Liefede and Herbison [26], Suckling et al. [25], and Dummer et al. [27] when many permanent teeth would not have erupted. In contrast, maxillary incisors are the most affected in the surveys conducted by Suckling et al. [25], Dummer et al. [27], Hoffman et al. [28], Angelillo et al., [21] and Rugg-Gunn et al. [12]. 

It was shown that the degree of enamel alterations varied depending on the kind of tooth and was more pronounced in locations with higher fluoride levels compared to those with lower fluoride levels. These data suggest a correlation between the alterations in enamel and the concentrations of drinking water [29]. 

The growing accessibility of dental products containing fluoride has sparked renewed interest in the study of dental fluorosis epidemiology. The occurrence of dental fluorosis is most closely associated with the overall accumulation of fluoride exposure throughout the development of teeth. Fluoride supplements may add to the overall fluoride exposure in children. If the total fluoride exposure to growing teeth is too high, it can lead to fluorosis, which causes defects in non-fluoridated locations and the severity of the defect worsens in fluoridated areas [8]. 

Limitations

The current research focused on certain villages and only included school children aged 12-15 years. Therefore, a comprehensive epidemiological investigation of the Fazilka district is necessary to provide a complete understanding of the problem's magnitude.

## Conclusions

The water in all the examined communities had a fluoride content ranging from 0.5 ppm to 2.0 ppm. Even when fluoride concentrations are low, the presence of an enamel defect in at least one tooth suggests that factors such as climate and the amount of water consumed have a role in addition to the concentration of fluoride in drinking water. Another contributing aspect might be the growing accessibility of dental products that include fluoride.

Fluoride supplements may add to the overall amount of fluoride that the growing teeth are exposed to, regardless of whether the location has naturally occurring fluoride or fluoride is added to the water supply. The prevailing enamel defect seen was diffuse opacity, with a score of 4, and it affected at least two-thirds of the tooth surface. Given that the current research focused on certain villages in the Fazilka area and only included school children aged 12-15 years, the findings indicate the need for a comprehensive epidemiological investigation of the whole Punjab state to have a more accurate understanding of the problem's magnitude. Given the negative impact of consuming water with excessive fluoride on oral health, it is crucial to prioritize the development of uncomplicated, efficient, and affordable de-fluoridation methods that are appropriate and acceptable to rural communities in high fluoride areas of Fazilka district. This becomes particularly important if there are no other accessible sources of drinking water with low fluoride content.

## References

[1] Hussein NN, Majid ZA, Mutalib KA, Abdullah F, Abang A, Wan MN (1999). Prevalence of developmental defects of enamel among 16 year old children in Malaysia. Annal Dent Univ Malaya.

[2] Dini EL, Holt RD, Bedi R (2000). Prevalence of caries and developmental defects of enamel in 9-10 year old children living in areas in Brazil with differing water fluoride histories. Br Dent J.

[3] Khan A, Moola MH, Cleaton-Jones P (2005). Global trends in dental fluorosis from 1980 to 2000: a systematic review. SADJ.

[4] Ramesh V, Nagesh L (2011). Assessment of fluoride concentration in drinking water and developmental defects of enamel in 14-15 years old school going children in villages of davangere taluk. J Indian Assoc Public Health Dent.

[5] Saravanan S, Kalyani C, Vijayarani M (2008). Prevalence of dental fluorosis among primary school children in rural areas of Chidambaram taluk, Cuddalore district, Tamil Nadu, India. Indian J Community Med.

[6] Whitford GM (1994). Intake and metabolism of fluoride. Adv Dent Res.

[7] Horowitz HS (1989). Fluoride and enamel defects. Adv Dent Res.

[8] Den Besten PK (1999). Mechanism and timing of fluoride effects on developing enamel. J Public Health Dent.

[9] Suckling GW (1989). Developmental defects of enamel--historical and present-day perspectives of their pathogenesis. Adv Dent Res.

[10] Ramesh G, Nagarajappa R, Raghunath V, Manohar R (2011). Developmental defects of enamel in children of Davangere District and their relationship to fluoride levels in drinking water. Asia Pac J Public Health.

[11] Clarkson J, O'Mullane D (1989). A modified DDE index for use in epidemiological studies of enamel defects. J Dent Res.

[12] Rugg-Gunn AJ, al-Mohammadi SM, Butler TJ (1997). Effects of fluoride level in drinking water, nutritional status, and socio-economic status on the prevalence of developmental defects of dental enamel in permanent teeth in Saudi 14-year-old boys. Caries Res.

[13] Chandrashekar J, Anuradha KP (2004). Prevalence of dental fluorosis in rural areas of Davangere, India. Int Dent J.

[14] Gopalakrishnan P, Vasan RS, Sarma PS, Nair KS, Thankappan KR (1999). Prevalence of dental fluorosis and associated risk factors in Alappuzha district, Kerala. Natl Med J India.

[15] Subbareddy VV, Tewari A (1985). Enamel mottling at different levels of fluoride in drinking water: in an endemic area. J Indian Dent Assoc.

[16] Narbutaite J, Milciuviene S, Larsen MJ (2000). Dental fluorosis and dental caries among 12-year-old Lithuanian children in low and high fluoride areas. Caries Res.

[17] Agarwal R, Chauhan SS (2014). The status of groundwater fluoride in Rajasthan: a case study of Devli tehsil, Tonk district. Int J Geol Earth Environ Sci.

[18] Venkateswarlu P, Rao DN, Rao KR (1952). Studies in endemic fluorosis: Visakhapatnam and suburban areas; fluorine, mottled enamel and dental caries. Indian J Med Res.

[19] Brouwer ID, DeBruin A, Dirks OB, Hautvast JG (1988). Unsuitability of World Health Organization guidelines for fluoride concentrations in drinking water in Senegal. Lancet.

[20] Nanda RS, Zipkin I, Doyle J, Horowitz HS (1974). Factors affecting the prevalence of dental fluorosis in Lucknow, India. Arch Oral Biol.

[21] Angelillo IF, Romano F, Fortunato L, Montanaro D (1990). Prevalence of dental caries and enamel defects in children living in areas with different water fluoride concentrations. Community Dent Health.

[22] Nunn JH, Rugg-Gunn AJ, Ekanayake L, Saparamadu KD (1994). Prevalence of developmental defects of enamel in areas with differing water fluoride levels and socio-economic groups in Sri Lanka and England. Int Dent J.

[23] Bardsen A, Klock KS, Bjorvatn K (1999). Dental fluorosis among persons exposed to high- and low-fluoride drinking water in western Norway. Community Dent Oral Epidemiol.

[24] Warnakulasuriya KA, Balasuriya S, Perera PA, Peiris LC (1992). Determining optimal levels of fluoride in drinking water for hot, dry climates--a case study in Sri Lanka. Community Dent Oral Epidemiol.

[25] Suckling GW, Pearce EI (1984). Developmental defects of enamel in a group of New Zealand children: their prevalence and some associated etiological factors. Community Dent Oral Epidemiol.

[26] de Liefde B, Herbison GP (1985). Prevalence of developmental defects of enamel and dental caries in New Zealand children receiving differing fluoride supplementation. Community Dent Oral Epidemiol.

[27] Dummer PM, Kingdon A, Kingdon R (1986). Distribution of developmental defects of tooth enamel by tooth-type in 11-12-year-old children in South Wales. Community Dent Oral Epidemiol.

[28] Hoffman MP, Cutress TW, Tomiki S (1988). Prevalence of developmental defects of enamel in children in the Kingdom of Tonga. N Z Dent J.

[29] Mahantesha T, Dixit UB, Nayakar RP, Ashwin D, Ramagoni NK, Kamavaram Ellore VP (2016). Prevalence of dental fluorosis and associated risk factors in Bagalkot District, Karnataka, India. Int J Clin Pediatr Dent.

